# Dynamics of a delayed rumor spreading model with discontinuous threshold control

**DOI:** 10.1016/j.heliyon.2022.e11231

**Published:** 2022-10-24

**Authors:** Chunru Li, Zujun Ma, Yuanyuan Wang

**Affiliations:** a Business School, Changshu Institute of Technology, Changshu, 215500, China; b School of Economics and Management, Southwest Jiaotong University, Chengdu, 610031, China

**Keywords:** Delay, Rumor spreading, Hopf bifurcation, Discontinuous control

## Abstract

In this paper, we studied a delayed rumor spreading model with discontinuous threshold control. First, we studied the existence of equilibria of the subsystem. Regarding the delay as bifurcating parameter, the local asymptotic stability and Hopf bifurcation of the positive equilibrium are discussed by analyzing the corresponding characteristic equations of linearized systems. Then, we studied the existence of the sliding mode and analyzed the existence of the tangent equilibria, boundary equilibria, regular equilibria, and the stability of the pseudo-equilibrium. Finally, we provide some numerical simulations to verify the theoretical results.

## 1 Introduction

Since the dawn of human civilization, rumors have consistently played a vital part in the social life of people, and they are a phenomenon that cannot be ignored. In essence, rumors are unsubstantiated stories or reports that circulate within a community, typically by word of mouth or social media, and are accepted as facts, despite the fact that their original source may be unknown. As information technology continues to advance, there has been a rise in interest in discussing social networks as a new medium for communication and social bonding. Currently, rumors are spreading more quickly than at any other time in history. People experience psychological panic as a result of it, and they suffer significant economic losses [1], [2]. For instance, when there was a nuclear leakage disaster in Japan, thousands of Chinese people made the error of thinking that buying iodized salt would protect them from nuclear radiation. This led to social panics and a lack of availability of table salt. It is therefore extremely important to conduct a comprehensive investigation into the rule of rumor propagation, in order to reduce rumor propagation while maintaining societal stability and security.

Understanding the characteristics of rumor dissemination and how it spreads can lead to more effective measures to preventing the spread of rumor. For this reason, the mathematical model, and in particular the epidemic model, is commonly utilized in the research of rumor dissemination in social networks. The mechanism by which rumors propagate on the web is strikingly similar to that by which infectious diseases spread. [3], [4], [5], [6], [7]. Daley and Kendall [8] first presented the DK model of rumor propagation. Moreno et al. studied [9] the stochastic version of the MK model on scale-free networks, by means of Monte Carlo simulations. Borge-Holthoefer et al. [10] introduced two mechanisms with the aim of filling the gap between theoretical and experimental results.

Undoubtedly, understanding how to effectively stop the spread of rumors is a crucial skill for preserving social harmony. Therefore, external controls on rumor spreading have been increasingly studied by academics, as have the norms of information distribution. Zhu [11] looked into how to stop rumors from spreading in online social networks. Zhao et al. [12] introduced a propagation force into the analysis of rumor propagation and discussed rumor control strategies. Zhu et al. [13] introduced a new delayed SIR (susceptible-propagating-recovery) epidemic-like rumor transmission model, which can be used in either a homogeneous or a heterogeneous network, and a forced silence function was introduced to discuss the control of the model. Zhu and Wang [14] studied a SAIR (susceptible-indifferent-propagating-recovery) rumor spreading model. We refer readers to refs. [15], [16], [17], [18] as some other related works on rumor models with control strategies.

It is noted that the control function of most models is applied at t=0. We point out that setting the beginning of control at t=0 is a very unrealistic assumption. With this in mind, a threshold control policy may be a better control strategy, which has been applied in an endemic model and a predator–prey model (see [19], [20], [21], [22]).

The following is the structure of the paper: In Section 3, some preliminaries are given. In Section 4, we conduct an analysis of the characteristic equations that correspond to each variable and explore the local stability as well as Hopf bifurcation. In Section 5, we talk about whether or not the system has a sliding domain. In Section 6, we will explain the main theoretical results through the use of numerical simulations that we will do. After then, the final portion of Section 7, which is a discussion, is presented.

## 2 Rumor spreading model formulation

We consider the threshold policy in a rumor spreading model. We divide the total people into three classes: the rumor-susceptible individuals S(t), who represent those unaware of the rumor; the rumor-propagating individuals I(t), who stand for those who believe and spread the rumor; the rumor-recovery individuals R(t), representing those who know the rumor but have ceased communicating it after meeting somebody already informed.

In the model, we assume that Λ is the constant rate of immigration of the rumor-susceptible individuals, and *β* is the contact rate of the rumor-susceptible individuals and the rumor-propagating individuals, *μ* is the removal rate of the system, *α* is the contact rate of the rumor-propagating peoples and the rumor-recovery peoples, that is, if the rumor-propagating peoples will become the rumor-recovery individuals after contacting with a rumor-recovery individual who tell the truth of the rumor. *τ* is the time delay from the rumor-propagating individuals to the rumor-recovery individuals.

In addition, we consider the threshold policy in the model: if the proportion of the rumor-propagating peoples is below the critical level $I_{th}$, the control is not applied, whereas, once the ratio of the rumor-propagating peoples increases and reaches a certain level $I_{th}$, the control strategy is implemented.

**Figure 1:**
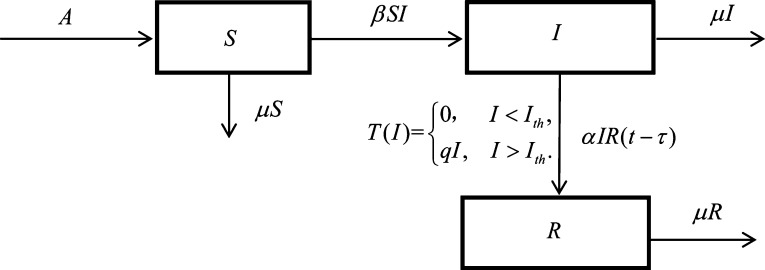
Organizational framework for the diffusion of rumors.

All the above parameters are all assumed positive. Fig. 1 depicts the model for the rumor spreading process based on the above facts.

In light of the above, the dynamics are governed by the system of nonlinear ordinary differential equations shown below.

(1) $$\left\{ \begin{array}{c} \frac{dS}{\mathrm{dt}}=\Lambda -\beta SI-\mu S,\\ \frac{dI}{\mathrm{dt}}=\beta SI-\alpha IR(t-\tau )-\mu I-T(I),\\ \frac{dR}{\mathrm{dt}}=\alpha IR(t-\tau )-\mu R+T(I), \end{array}\right.$$

where T(I) is a government control function, which is a threshold control as follows

$$T(I)=\left\{ \begin{array}{cc} 0,\; & I<I_{th},\;\\ qI,\; & I>I_{th},\; \end{array}\right.$$

where $I_{th}$ is a threshold value. This means that only when $I>I_{th}$ will the control be applied.

To best our knowledge, few researchers have examined a model of delayed rumor propagation with discontinuous threshold control. We shall examine the existence of the equilibria, its stability and bifurcation in this paper.

## 3 Preliminaries

First, we introduce some helpful attributes and definitions for Filippov systems [23].

Letting $H(Z)=I-I_{th}$ with vector $Z={\left(S,I,R\right)}^{T}$, and

$${F_{G^{1}}(Z)=(\Lambda -\beta SI-\mu S,\beta SI-\alpha IR(t-\tau )-\mu I,\alpha IR(t-\tau )-\mu R))}^{T},{F_{G^{2}}(Z)=(\Lambda -\beta SI-\mu S,\beta SI-\alpha IR(t-\tau )-qI,\alpha IR(t-\tau )-\mu R+qI))}^{T}.$$

Consequently, system (1) can be expressed as the Filippov system

(2) $$\frac{\partial Z}{\partial t}=\left\{ \begin{array}{cc} F_{G^{1}}(Z),\; & Z\in G_{1}:=\{Z|H(Z)<0\},\;\\ F_{G^{2}}(Z),\; & Z\in G_{2}:==\{Z|H(Z)>0\}.\; \end{array}\right.$$

In addition, $\Sigma_{s}=\{Z|H(Z)=0\}$ describes the discontinuity border dividing the two regions $G_{1}$ and $G_{2}$. The Filippov system (2) defined in $G_{1}$ is called $S_{1}$ while that defined in $G_{2}$ is called $S_{2}$.

Let

$$\sigma (Z)=\langle H_{Z}(Z),F_{G_{1}}(Z)\rangle \langle H_{Z}(Z),F_{G_{2}}(Z)\rangle ,$$

where 〈⋅〉 represents the conventional scalar product. Following, we shall employ the notation $F_{G_{i}}\cdot H(z)=\langle H_{Z}(Z),F_{G_{i}}(Z)\rangle$ for i=1,2. Thus, the sliding mode domain can be defined as

$$\Sigma_{S}=\{Z\in \Sigma |\sigma (Z)<0\}.$$

Throughout the study, all forms of Filippov equilibria are defined as the following [24], [25], [26].

Definition 3.1 If $F_{G^{1}}(Z^{⁎})=0$, $H(Z^{⁎})<0$ or $F_{G^{2}}(Z^{⁎})=0$, $H(Z^{⁎})>0$, then $Z^{⁎}$ is called a regular equilibrium of system (2), and if $F_{G^{1}}(Z^{⁎})=0$, $H(Z^{⁎})>0$ or $F_{G^{2}}(Z^{⁎})=0$, $H(Z^{⁎})<0$, then $Z^{⁎}$ is called a virtual equilibrium of system (2).

Definition 3.2 A point $Z^{⁎}$ is called a pseudo-equilibrium if it is an equilibrium of the sliding mode of system (2), that is, $(1-\lambda )F_{G_{1}}(Z^{⁎})+\lambda F_{G_{2}}(Z^{⁎})=0$, $H(Z^{⁎})=0$, and 0<λ<1, where

$$\lambda =\frac{\langle H_{Z}(Z),F_{G_{1}}(Z)\rangle }{\langle H_{Z}(Z),F_{G_{1}}(Z)-F_{G_{2}}(Z)\rangle }.$$

Definition 3.3 The tangent equilibrium $Z^{⁎}$ of system (2) is that $Z^{⁎}\in \Sigma_{s}$ and $\langle H_{Z}(Z^{⁎}),F_{G_{1}}(Z^{⁎})\rangle =0$ or $\langle H_{Z}(Z^{⁎}),F_{G_{2}}(Z^{⁎})\rangle =0$.

Definition 3.4 The boundary equilibrium $Z^{⁎}$ of system (2) is that $F_{G_{1}}(Z^{⁎})=0$, $H_{Z}(Z^{⁎})=0$ or $F_{G_{2}}(Z^{⁎})=0$, $H_{Z}(Z^{⁎})=0$.

## 4 Dynamics of subsystem

### 4.1 Dynamics of subsystem $S_{1}$

For subsystem $S_{1}$, the equilibria satisfy

(3) $$\left\{ \begin{array}{c} \Lambda -\beta SI-\mu S=0,\;\\ \beta SI-\alpha IR-\mu I=0,\;\\ \alpha IR-\mu R=0.\; \end{array}\right.$$

Obviously, subsystem $S_{1}$ has a equilibrium $P_{S_{1}}^{1}=(\frac{A}{\mu },0,0)$, which is always a regular equilibrium, and it implies that both rumor-propagating individuals and rumor-recovery individuals are extinct.

If R=0, from the second equation of (3), we can obtain that $S=\frac{\mu }{\beta }$. Then substitute it into the first equation of (3), we have $I=\frac{\Lambda \beta -\mu^{2}}{\beta \mu }$. Therefore, if $\mu^{2}<\Lambda \beta$, then the system has a equilibrium $P_{S_{1}}^{2}=(\frac{\mu }{\beta },\frac{\Lambda \beta -\mu^{2}}{\beta \mu },0)$, which implies that the rumor-recovery individuals are extinct.

For the positive equilibrium, from the third equation of (3), we have $I=\frac{\mu }{\alpha }$. Substitute it into the first equation of (3), one obtains that $S=\frac{\Lambda \alpha }{\mu (\alpha +\beta )}$. Then according to the second equation of (3), we have $R=\frac{\beta \Lambda \alpha -\mu^{2}(\alpha +\beta )}{\alpha \mu (\alpha +\beta )}$. Hence, if $\beta \Lambda \alpha -\mu^{2}(\alpha +\beta )>0$, then subsystem $S_{1}$ has a positive equilibrium $P_{S_{1}}^{3}=(S_{S_{1}}^{⁎},I_{S_{1}}^{⁎},R_{S_{1}}^{⁎})=(\frac{\Lambda \alpha }{\mu (\alpha +\beta )},\frac{\mu }{\alpha },\frac{\beta \Lambda \alpha -\mu^{2}(\alpha +\beta )}{\alpha \mu (\alpha +\beta )})$.

Theorem 4.1 *For system* $S_{1}$ *, we have*

*(i) when* $\Lambda \beta <\mu^{2}$ *, system* $S_{1}$ *has only an equilibrium* $P_{S_{1}}^{1}=(\frac{A}{\mu },0,0)$ *which is locally asymptotically stable for any* τ≥0 *;*

*(ii) when* $\mu^{2}<\Lambda \beta <\mu^{2}+\frac{\mu^{2}\beta }{\alpha }$ *, the equilibrium* $P_{S_{1}}^{1}=(\frac{A}{\mu },0,0)$ *is unstable and the equilibrium* $P_{S_{1}}^{2}=(\frac{\mu }{\beta },\frac{\Lambda \beta -\mu^{2}}{\beta \mu },0)$ *is locally asymptotically stable for any* τ≥0 *;*

*(iii) When* $\Lambda \beta >\mu^{2}+\frac{\mu^{2}\beta }{\alpha }$ *, the equilibria* $P_{S_{1}}^{1}$ *and* $P_{S_{1}}^{2}$ *are both unstable, and system has a unique positive equilibrium* $P_{S_{1}}^{3}=(S_{S_{1}}^{⁎},I_{S_{1}}^{⁎},R_{S_{1}}^{⁎})=(\frac{\Lambda \alpha }{\mu (\alpha +\beta )},\frac{\mu }{\alpha },\frac{\beta \Lambda \alpha -\mu^{2}(\alpha +\beta )}{\alpha \mu (\alpha +\beta )})$ *, which is locally asymptotically stable with* τ=0 *.*

Proof (i) For the equilibrium $P_{S_{1}}^{1}=(\frac{\Lambda }{\mu },0,0)$, $J_{\left(\frac{\Lambda }{\mu },0,0\right)}=\left(\begin{array}{ccc} -\mu \; & -\frac{\beta \Lambda }{\mu }\; & 0\\ 0\; & \frac{\beta \Lambda }{\mu }-\mu \; & 0\\ 0\; & 0\; & -\mu \end{array}\right)$. If $\Lambda \beta <\mu^{2}$, then $\lambda_{2}=\frac{\beta \Lambda }{\mu }-\mu <0$, and $\lambda_{1}=-\mu <0$, $\lambda_{3}=-\mu <0$, so $P_{S_{1}}^{1}$ is locally asymptotically stable for any τ≥0, which means that rumor is wiped out in the social.

(ii) For the equilibrium $P_{S_{1}}^{2}=(\frac{\mu }{\beta },\frac{\Lambda \beta -\mu^{2}}{\beta \mu },0)$,

$$J_{\left(\frac{\mu }{\beta },\frac{\Lambda \beta -\mu^{2}}{\beta \mu },0\right)}=\left(\begin{array}{ccc} -\frac{\Lambda \beta -\mu^{2}}{\mu }\; & -\mu \; & 0\\ -\frac{\Lambda \beta -\mu^{2}}{\mu }\; & 0\; & -\alpha \frac{\Lambda \beta -\mu^{2}}{\beta \mu }\\ 0\; & 0\; & \alpha \frac{\Lambda \beta -\mu^{2}}{\beta \mu }-\mu \end{array}\right),$$

and the corresponding characteristic equation is

(4) $$(\lambda -\alpha \frac{\Lambda \beta -\mu^{2}}{\beta \mu }+\mu )(\lambda^{2}+\frac{\Lambda \beta -\mu^{2}}{\mu }\lambda +\Lambda \beta -\mu^{2})=0.$$

Obviously, all roots of Eq. (4) are negative. Therefore, $P_{S_{1}}^{2}$ is locally asymptotically stable.

(iii) When $\Lambda \beta >\mu^{2}+\frac{\mu^{2}\beta }{\alpha }$, for the equilibrium $P_{S_{1}}^{3}=(S_{S_{1}}^{⁎},I_{S_{1}}^{⁎},R_{S_{1}}^{⁎})$ the corresponding characteristic equation is

(5) $$\lambda^{3}+A_{1}\lambda^{2}+A_{2}\lambda +A_{3}+(A_{4}\lambda^{2}+A_{5}\lambda +A_{6})e^{-\lambda \tau }=0,$$

where

$$A_{1}=2\mu +\beta I_{S_{1}}^{⁎},A_{2}=\mu (\beta I_{S_{1}}^{⁎}+\mu )+I_{S_{1}}^{⁎}S_{S_{1}}^{⁎}\beta^{2},A_{3}=\beta^{2}S_{S_{1}}^{⁎}I_{S_{1}}^{⁎}\mu ,A_{4}=-\alpha I_{S_{1}}^{⁎},A_{5}=\alpha^{2}R_{S_{1}}^{⁎}I_{S_{1}}^{⁎}-\alpha I_{S_{1}}^{⁎}(\beta I_{S_{1}}^{⁎}+\mu ),A_{6}=(\beta I_{S_{1}}^{⁎}+\mu )\alpha^{2}R_{S_{1}}^{⁎}I_{S_{1}}^{⁎}-\beta^{2}\alpha S_{S_{1}}^{⁎}I_{S_{1}}^{⁎2}.$$

When τ=0, the characteristic equation reduces to

$$\lambda^{3}+(A_{1}+A_{4})\lambda^{2}+(A_{2}+A_{5})\lambda +A_{3}+A_{6}=0.$$

By a direction calculation, we obtain

$$A_{1}+A_{4}=\beta I_{S_{1}}^{⁎}+2\mu -\alpha I_{S_{1}}^{⁎}=\frac{\alpha \mu +\beta \mu }{\alpha }>0,A_{3}+A_{6}=-\frac{\alpha \mu^{3}+\beta \mu^{3}-\Lambda \alpha \beta \mu }{\alpha }>0,(A_{1}+A_{4})(A_{2}+A_{5})-(A_{3}+A_{6})=\frac{\Lambda \beta^{2}\mu }{\alpha }>0.$$

Therefore, when τ=0 all the roots of Eq. (5) have a negative real part. According to the Routh-Hurwitz criterion, the equilibrium $P_{S_{1}}^{3}$ is locally asymptotically stable when τ=0. □

Remark 4.1 $P_{S_{1}}^{1}$ is stable implying that rumor does not spread, and rumor-propagating and rumor-recovery peoples are both extinct. $P_{S_{1}}^{2}$ and $P_{S_{1}}^{3}$ are stable implying that rumor always exists and maintain at a constant rate.

When τ>0, let λ=iω(ω>0) be a solution of Eq. (5). Separating real and imaginary parts, we have

(6) $$\left\{ \begin{array}{cc} \; & -\omega^{3}+A_{2}\omega =(-A_{4}\omega^{2}+A_{6})\sin\omega \tau -A_{5}\omega \cos\tau ,\;\\ \; & -A_{1}\omega^{2}+A_{3}=-A_{5}\omega \sin\omega \tau -(-A_{4}\omega^{2}+A_{6})\cos\omega \tau ,\; \end{array}\right.$$

which leads to

(7) $$\omega^{6}+B_{1}\omega^{4}+B_{2}\omega^{2}+B_{3}=0,$$

where

$$B_{1}=A_{1}^{2}-2A_{2}-A_{4}^{2},B_{2}=A_{2}^{2}-2A_{1}A_{3}+2A_{4}A_{6}-A_{5}^{2},B_{3}=A_{3}^{2}-A_{6}^{2}.$$

Let $v=\omega^{2}$, we have

$$v^{3}+B_{1}v^{2}+B_{2}v+B_{3}=0.$$

Denote

(8) $$h(v)=v^{3}+B_{1}v^{2}+B_{2}v+B_{3}.$$

Obviously, we have

$$h^{\prime }(v)=3v^{2}+2B_{1}v+B_{2}.$$

Denote

$$\Delta =4B_{1}^{2}-12B_{2}.$$

For the roots of (8), we have the following results.

Lemma 4.1 *(i) Eq.*(8)*doesn't have positive root if*Δ≤0*,*$B_{3}\geq 0$*, or*Δ>0*,*$v^{⁎}=\frac{-2B_{1}+\sqrt{\Delta }}{6}<0$*,*$B_{3}>0$*, or*Δ>0*,*$v^{⁎}>0$$B_{3}\geq 0$*,*$h(v^{⁎})>0$*.*

*(ii) Eq.*(8)*doesn't have positive root if*Δ>0*,*$v^{⁎}>0$*,*$B_{3}\geq 0$*,*$h(v^{⁎})<0$*or*$B_{3}<0$*.*

Suppose that Eq. (7) has one positive root $\omega_{10}=\sqrt{v_{0}}$. From (6), we have

$$\cos\omega \tau =\frac{\left(-A_{4}\omega_{10}^{2}+A_{6})(-A_{1}\omega_{10}^{2}+A_{3})+A_{5}\omega_{10}(-\omega_{10}^{3}+A_{2}\omega_{10}\right)}{{\left(-A_{4}\omega_{10}^{2}+A_{6}\right)}^{2}+A_{5}^{2}\omega_{10}^{2}}.$$

Therefore, the delay's critical value is

(9) $$\tau_{j}=\frac{1}{\omega_{10}}(arccos\frac{\left(-A_{4}\omega_{10}^{2}+A_{6})(-A_{1}\omega_{10}^{2}+A_{3})+A_{5}\omega_{10}(-\omega_{10}^{3}+A_{2}\omega_{10}\right)}{{\left(-A_{4}\omega_{10}^{2}+A_{6}\right)}^{2}+A_{5}^{2}\omega_{10}^{2}}+2j\pi ),j=0,1,2,\cdots .$$

It should be noted that the roots of Eq. (5) with $\tau =\tau_{0}$ are $\pm i\omega_{10}$. As a result of doing a first-order derivative of Eq. (5) with respect to *τ*, we have

$${\left(\frac{d\lambda }{d\tau }\right)}^{-1}=\frac{3\lambda^{2}+2A_{1}\lambda +A_{2}}{\lambda^{4}+A_{1}\lambda^{3}+A_{2}\lambda^{2}+A_{3}\lambda }+\frac{2A_{4}\lambda +A_{5}}{A_{4}\lambda^{3}+A_{5}\lambda^{2}+A_{6}\lambda }-\frac{\tau }{\lambda },$$

which yields to

$$Re{\left(\frac{d\lambda }{d\tau }\right)}^{-1}{|}_{\lambda =i\omega_{10}}=\frac{3\omega_{0}^{4}+(2A_{1}^{2}-4A_{2}-2A_{4}^{2})\omega_{10}^{2}}{{\left(A_{4}\omega_{10}^{2}-A_{6}\right)}^{2}+A_{5}^{2}\omega_{10}^{2}}\;\;+\frac{A_{2}^{2}+2A_{4}A_{6}-2A_{1}A_{3}-A_{5}^{2}}{{\left(A_{4}\omega_{10}^{2}-A_{6}\right)}^{2}+A_{5}^{2}\omega_{10}^{2}}=\frac{h^{\prime }(\omega_{10}^{2})}{{\left(A_{4}\omega_{10}^{2}-A_{6}\right)}^{2}+A_{5}^{2}\omega_{10}^{2}}.$$

Based on the above discussions, we have the following conclusion.

Theorem 4.2 *If*$\Lambda \beta >\mu^{2}+\frac{\mu^{2}\beta }{\alpha }$*holds, then these conclusions hold.*

*(1) If Eq.*(7)*has no positive real root, then*$P_{S_{1}}^{3}$*is locally asymptotically stable for all*τ>0*;*

*(2) If Eq.*(7)*has a positive root, then*$P_{S_{1}}^{3}$*is locally asymptotically stable when*$\tau \in [0,\tau_{0})$*and unstable when*$\tau \in (\tau_{0},+\infty )$*, which means that system*$S_{1}$*has a Hopf bifurcation at*$P_{S_{1}}^{3}$*when*$\tau =\tau_{0}$*.*

### 4.2 Dynamics of subsystem $S_{2}$

Theorem 4.3 *For system* $S_{2}$ *, we have*

*(i) when* $\Lambda \beta <\mu^{2}+q\mu$ *, system* $S_{2}$ *has only an equilibrium* $P_{S_{2}}^{1}=(\frac{A}{\mu },0,0)$ *, which is always a virtual equilibrium for* $I_{th}>0$ *;*

*(ii) when* $\Lambda \beta >\mu^{2}+q\mu$ *system* $S_{2}$ *has a unique positive equilibrium* $P_{S_{2}}^{2}=(S_{S_{2}}^{⁎},I_{S_{2}}^{⁎},R_{S_{2}}^{⁎})$ *with* $S_{S_{2}}^{⁎}=\frac{\alpha R_{S_{2}}^{⁎}+\mu +q}{\beta }$ *,* $I_{S_{2}}^{⁎}=\frac{\mu R_{S_{2}}^{⁎}}{\alpha R_{S_{2}}^{⁎}+q}$ *, where*

$$R^{⁎}=\frac{-C_{2}+\sqrt{C_{2}^{2}-4C_{1}C_{2}}}{2C_{1}},$$

*and*

$$C_{1}=\mu \alpha (\alpha +\beta ),C_{2}=\alpha \mu^{2}+\beta \mu^{2}-\Lambda \alpha \beta +2\alpha \mu q+\beta \mu q,C_{3}=q(\mu^{2}+q\mu -\Lambda \beta ).$$

Proof For system $S_{2}$, $P_{S_{2}}^{1}=(\frac{A}{\mu },0,0)$ is obvious a virtual equilibrium, and $S_{S_{2}}^{⁎},I_{S_{2}}^{⁎}$, and $R_{S_{2}}^{⁎}$ satisfy

(10) $$\left\{ \begin{array}{c} \Lambda -\beta SI-\mu S=0,\;\\ \beta S-\alpha R-\mu -q=0,\;\\ \alpha IR-\mu R+qI=0.\; \end{array}\right.$$

From the second equation of Eq. (10), we have

(11) $$S=\frac{\alpha R+\mu +q}{\beta }.$$

According to the third equation of Eq. (10), we have

(12) $$I=\frac{\mu R}{\alpha R+q}.$$

Then, substituting (11) and (12) into the third equation of (10), we get

(13) $$C_{1}R^{2}+C_{2}R+C_{3}=0.$$

When $\Lambda \beta >\mu^{2}+q\mu$, $C_{3}=q(\mu^{2}+q\mu -\Lambda \beta )<0$. Therefore, Eq. (13) has a unique positive root $R_{S_{2}}^{⁎}$. Then, by the third equation of (10), we have $I_{S_{2}}^{⁎}=\frac{\mu R_{S_{2}}^{⁎}}{\alpha R_{S_{2}}^{⁎}+q}$, and by the second equation of (10), $S_{S_{2}}^{⁎}=\frac{\mu +q+\alpha R_{S_{2}}^{⁎}}{\beta }$. Consequently, system $S_{2}$ has a unique positive equilibrium $P_{S_{2}}^{2}=(S_{S_{2}}^{⁎},I_{S_{2}}^{⁎},R_{S_{2}}^{⁎})$. □

Remark 4.2 In addition, we have

$$I_{S_{2}}^{⁎}=\frac{\mu R_{S_{2}}^{⁎}}{\alpha R_{S_{2}}^{⁎}+q}<\frac{\mu }{\alpha }=I_{S_{1}}^{⁎}.$$

At $P_{S_{2}}^{2}=(S_{S_{2}}^{⁎},I_{S_{2}}^{⁎},R_{S_{2}}^{⁎})$, the characteristic equation is

(14) $$\lambda^{3}+D_{1}\lambda^{2}+D_{2}\lambda +D_{3}+(D_{4}\lambda^{2}+D_{5}\lambda +D_{6})e^{-\lambda \tau }=0,$$

where

(15) $$D_{1}=2\mu +\beta I_{S_{2}}^{⁎},D_{2}=\beta^{2}S_{S_{2}}^{⁎}I_{S_{2}}^{⁎}+\beta \mu I_{S_{2}}^{⁎}+\mu^{2},D_{3}=\beta^{2}\mu S_{S_{2}}^{⁎}I_{S_{2}}^{⁎},D_{4}=-\alpha I_{S_{2}}^{⁎},D_{5}=\alpha I_{S_{2}}^{⁎}(-I_{S_{2}}^{⁎}\beta +\alpha R_{S_{2}}^{⁎}-\mu +q),D_{6}=(\alpha R_{S_{2}}^{⁎}+q)(\beta I_{S_{2}}^{⁎}+\mu )-\beta^{2}S_{S_{2}}^{⁎}I_{S_{2}}^{⁎}.$$

When τ=0, the characteristic equation reduces to

$$\lambda^{3}+(D_{1}+D_{4})\lambda^{2}+(D_{2}+D_{5})\lambda +D_{3}+D_{6}=0.$$

According to (15), by a direct calculation, we have

(16) $$D_{3}+D_{6}=\beta^{2}\mu S_{S_{2}}^{⁎}I_{S_{2}}^{⁎}+\alpha I_{S_{2}}^{⁎}(\alpha R_{S_{2}}^{⁎}+q)(\beta I_{S_{2}}^{⁎}+\mu )-\alpha \beta^{2}S_{S_{2}}^{⁎}I_{S_{2}}^{⁎2}=\frac{\mu^{2}}{{\left(q+R_{S_{2}}^{⁎}\alpha \right)}^{2}}(R_{S_{2}}^{⁎3}\alpha^{3}+\beta R_{S_{2}}^{⁎3}\alpha^{2}+2R_{S_{2}}^{⁎2}\alpha^{2}q+2\beta R_{S_{2}}^{⁎2}\alpha q+R_{S_{2}}^{⁎}\alpha q^{2}\;\;+\beta R_{S_{2}}^{⁎}\mu q+\beta R_{S_{2}}^{⁎}q^{2})>0,(D_{1}+D_{4})(D_{2}+D_{5})-(D_{3}+D_{6})=\frac{\mu^{2}(q+R_{S_{2}}^{⁎}\beta )}{{\left(q+R_{S_{2}}^{⁎}\alpha \right)}^{3}}(R_{S_{2}}^{⁎3}\alpha^{3}+\beta_{S_{2}}^{⁎3}\alpha^{2}+2_{S_{2}}^{⁎2}\alpha^{2}q+\mu R_{S_{2}}^{⁎2}\alpha^{2}+2\beta R_{S_{2}}^{⁎2}\alpha q+\beta \mu R_{S_{2}}^{⁎2}\alpha +R_{S_{2}}^{⁎}\alpha q^{2}+3\mu R_{S_{2}}^{⁎}\alpha q+\beta R_{S_{2}}^{⁎}q^{2}+2\beta \mu R_{S_{2}}^{⁎}q+2\mu q^{2})>0.$$

Therefore, all the roots of Eq. (14) have a negative real part. Then, according to the Routh-Hurwitz criterion, the equilibrium $P_{S_{2}}^{2}$ is locally asymptotically stable when τ=0.

Following is a discussion of *τ*'s effects. Assume λ=iω(ω>0) is a root of (14). Then, by separating the real and imagined components, we obtain

(17) $$\left\{ \begin{array}{cc} \; & -\omega^{3}+D_{2}\omega =(-D_{4}\omega^{2}+D_{6})\sin\omega \tau -D_{5}\omega \cos\tau ,\;\\ \; & -D_{1}\omega^{2}+D_{3}=-D_{5}\omega \sin\omega \tau -(-D_{4}\omega^{2}+D_{6})\cos\omega \tau ,\; \end{array}\right.$$

which leads to

(18) $$\omega^{6}+(D_{1}^{2}-2D_{2}-D_{4}^{2})\omega^{4}+(D_{2}^{2}-2D_{1}D_{3}+2D_{4}D_{6}-D_{5}^{2})\omega^{2}+D_{3}^{2}-D_{6}^{2}=0.$$

Let $v_{1}=\omega^{2}$, we have

$$v_{1}^{3}+(D_{1}^{2}-2D_{2}-D_{4}^{2})v_{1}^{2}+(D_{2}^{2}-2D_{1}D_{3}+2D_{4}D_{6}-D_{5}^{2})v_{1}+D_{3}^{2}-D_{6}^{2}=0.$$

Denote

$$h_{1}(v_{1})=v_{1}^{3}+(D_{1}^{2}-2D_{2}-D_{4}^{2})v_{1}^{2}+(D_{2}^{2}-2D_{1}D_{3}+2D_{4}D_{6}-D_{5}^{2})v_{1}+D_{3}^{2}-D_{6}^{2}.$$

Obviously, we have

$$h_{1}^{\prime }(v_{1})=3v_{1}^{2}+2(D_{1}^{2}-2D_{2}-D_{4}^{2})v_{1}+(D_{2}^{2}-2D_{1}D_{3}+2D_{4}D_{6}-D_{5}^{2}).$$

Lemma 4.2 *If* α<β *holds, then Eq.* (18) *has at least a positive root.*

Proof From (16), we know $D_{3}+D_{6}>0$. Under the condition α<β, we have

$$D_{3}-D_{6}=(\alpha -\beta )(R^{3}\alpha^{2}\mu^{2}+2R^{2}\alpha \mu^{2}q+R\mu^{2}q^{2})-\beta R\mu^{2}q(\mu +q)<0.$$

Therefore, we obtain $D_{3}^{2}-D_{6}^{2}<0$. Obviously, $\underset{v_{1}\to +\infty }{\lim}\;h_{1}(v_{1})=+\infty$. Hence, there is at least $v_{1}^{⁎}\in (0,\infty )$ such that $h_{1}(v_{1}^{⁎})=0$, which means that Eq. (18) has at least a positive root. □

Assume that Eq. (18) has one positive root $\omega_{20}=\sqrt{v_{1}^{⁎}}$. From (17), we have

$$\cos\omega \tau =\frac{\left(-D_{4}\omega_{0}^{2}+D_{6})(-D_{1}\omega_{0}^{2}+D_{3})+D_{5}\omega_{0}(-\omega_{0}^{3}+D_{2}\omega_{0}\right)}{{\left(-D_{4}\omega_{0}^{2}+D_{6}\right)}^{2}+D_{5}^{2}\omega_{0}^{2}}.$$

Therefore, the delay's crucial value is

(19) $$\tau_{2}^{j}=\frac{1}{\omega_{20}}(arccos\frac{\left(-D_{4}\omega_{20}^{2}+D_{6})(-D_{1}\omega_{20}^{2}+D_{3})+D_{5}\omega_{20}(-\omega_{20}^{3}+D_{2}\omega_{20}\right)}{{\left(-D_{4}\omega_{20}^{2}+D_{6}\right)}^{2}+D_{5}^{2}\omega_{20}^{2}}+2j\pi ),j=0,1,2,\cdots .$$

Differentiating Eq. (14) with regard to *τ* yields

$${\left(\frac{d\lambda }{d\tau }\right)}^{-1}=\frac{3\lambda^{2}+2D_{1}\lambda +D_{2}}{\lambda^{4}+D_{1}\lambda^{3}+D_{2}\lambda^{2}+D_{3}\lambda }+\frac{2D_{4}\lambda +D_{5}}{D_{4}\lambda^{3}+D_{5}\lambda^{2}+D_{6}\lambda }-\frac{\tau }{\lambda },$$

which yields to

(20) $$Re{\left(\frac{d\lambda }{d\tau }\right)}^{-1}{|}_{\lambda =i\omega_{20}}=\frac{3\omega_{20}^{4}+(2D_{1}^{2}-4D_{2}-2A_{4}^{2})\omega_{20}^{2}}{{\left(D_{4}\omega_{20}^{2}-D_{6}\right)}^{2}+D_{5}^{2}\omega_{20}^{2}}\;\;+\frac{D_{2}^{2}+2D_{4}D_{6}-2D_{1}D_{3}-D_{5}^{2}}{{\left(D_{4}\omega_{0}^{2}-A_{6}\right)}^{2}+A_{5}^{2}\omega_{20}^{2}}=\frac{h_{1}^{\prime }(\omega_{20}^{2})}{{\left(D_{4}\omega_{20}^{2}-D_{6}\right)}^{2}+D_{5}^{2}\omega_{20}^{2}}.$$

Based on the above discussions, we have the following result.

Theorem 4.4 *If*$\Lambda \beta >\mu^{2}+q\mu$*and*α<β*hold, then the following conclusions are true.*

*(i) The equilibrium*$P_{S_{2}}^{2}$*is locally asymptotically stable for*$\tau \in [0,\tau_{2}^{0})$*;*

*(ii) System*$S_{2}$*has a Hopf bifurcation at*$P_{S_{2}}^{2}$*when*$\tau =\tau_{2}^{0}$*.*

Remark 4.3 Under the conditions of Theorem 4.2 and Theorem 4.4, a stability switch may occur at the positive equilibria $P_{S_{1}}^{3}$ or $P_{S_{2}}^{2}$. We left it out and are only studying the Hopf bifurcation phenomenon here.

Remark 4.4 The system has a Hopf bifurcation implying that rumor spreads in a cyclically oscillating manner, which is harmful to the stability of the social realm.

## 5 Sliding domain and its dynamics

### 5.1 Equilibria of system (2)

In Definition 3.1-3.4, it is shown that the equilibria of the Filippov system (2) can be classified into several categories. We will abbreviate these equilibria as $E_{R}$, $E_{V}$, $E_{P}$, $E^{B}$, and $E^{T}$ for clarity.

**Tangent equilibrium:** By Definition 3.3, the tangent equilibrium $E_{T}$ satisfies equation

$$\left\{ \begin{array}{c} \beta SI-\alpha IR-\mu I-\varepsilon qI=0,\;\\ I=I_{th}.\; \end{array}\right.$$

Therefore, the tangent equilibria can be shown as

$$E_{T}=\{((S,I,R)|I=I_{th},\beta S-\alpha R=\mu +\varepsilon q\}.$$

**Boundary equilibrium:** By Definition 3.4, to find the boundary equilibrium, set

(21) $$\left\{ \begin{array}{c} \Lambda -\beta SI-\mu S=0,\;\\ \beta S-\alpha R-\mu -\varepsilon q=0,\;\\ \alpha IR-\mu R+\varepsilon qI=0,\;\\ I=I_{th}.\; \end{array}\right.$$

When ε=0, we have that if

$$\frac{\Lambda }{\beta I_{th}+\mu }=\frac{\mu }{\beta }$$

holds, then there exists a boundary equilibrium $E_{b}^{1}=(\frac{\mu }{\beta },I_{th},0)$. Otherwise, if $\frac{\mu }{\alpha }=I_{th}$, then the system has another boundary equilibrium

$$E_{b}^{2}=\left(\frac{\Lambda }{\beta I_{th}+\mu },I_{th},\frac{\beta \Lambda -\mu (\beta I_{th}+\mu )}{\alpha (\beta I_{th}+\mu )}\right).$$

When ε=1, from the first equation of Eq. (21) we have

(22) $$S=\frac{\Lambda }{\beta I_{th}+\mu }.$$

Substitute (22) into the second of (21), we obtain

$$R=\frac{\beta \Lambda -(\mu +q)(\beta I_{th}+\mu )}{\alpha (\beta I_{th}+\mu )}.$$

From the third equation of Eq. (21), we get

$$R=\frac{qI_{th}}{\mu -\alpha I_{th}}.$$

Therefore, if the following conditions hold,

$$\mu >\alpha I_{th},\beta \Lambda >(\mu +q)(\beta I_{th}+\mu ),\frac{\beta \Lambda -(\mu +q)(\beta I_{th}+\mu )}{\alpha (\beta I_{th}+\mu )}=\frac{qI_{th}}{\mu -\alpha I_{th}},$$

then system (2) has a boundary equilibrium

$$E_{b}^{2}=\left(\frac{\Lambda }{\beta I_{th}+\mu },I_{th},\frac{qI_{th}}{\mu -\alpha I_{th}}\right).$$

**Regular equilibrium:** To evaluate the regular and virtual equilibria of Filippov system (2), it is important to examine all of the subsystem $S_{1}$ and $S_{2}$ equilibria.

According to the above discussions, system $S_{1}$ has three equilibria, where $P_{S_{1}}^{1}=(\frac{A}{\mu },0,0)$ is always a regular equilibrium. The internal equilibria are $P_{S_{1}}^{2}$ and $P_{S_{1}}^{3}$, and they can be classified as follows.

(i) If $0<\frac{\Lambda \beta -\mu^{2}}{\beta \mu }<\min\left\{\frac{\mu }{\alpha },I_{th}\right\}$, then $P_{S_{1}}^{2}$ is a regular equilibrium, while $P_{S_{1}}^{3}$ does not exist;

(ii) if $I_{th}<\frac{\Lambda \beta -\mu^{2}}{\beta \mu }<\frac{\mu }{\alpha }$, then $P_{S_{1}}^{2}$ is a virtual equilibrium, while $P_{S_{1}}^{3}$ does not exist;

(iii) if $\frac{\mu }{\alpha }<\frac{\Lambda \beta -\mu^{2}}{\beta \mu }<I_{th}$, then $P_{S_{1}}^{2}$ and $P_{S_{1}}^{3}$ are both regular equilibrium;

(iv) if $\frac{\mu }{\alpha }<I_{th}<\frac{\Lambda \beta -\mu^{2}}{\beta \mu }$, then $P_{S_{1}}^{2}$ is a virtual equilibrium, while $P_{S_{1}}^{3}$ is a regular equilibrium;

(vi) if $I_{th}<\frac{\mu }{\alpha }<\frac{\Lambda \beta -\mu^{2}}{\beta \mu }$, then $P_{S_{1}}^{2}$ and $P_{S_{1}}^{3}$ are both virtual equilibria.

For subsystem $S_{2}$, $P_{S_{2}}^{1}$ is always a virtual equilibrium. It only contains a unique internal equilibrium $P_{S_{2}}^{2}=(S_{S_{2}}^{⁎},I_{S_{2}}^{⁎},R_{S_{2}}^{⁎})$, and it can be classified as follows.

(i) If $I_{S_{2}}^{⁎}<I_{th}$, then $P_{S_{2}}^{2}$ is a virtual equilibrium;
(ii) if $I_{S_{2}}^{⁎}>I_{th}$, then $P_{S_{2}}^{2}$ is a regular equilibrium.

Remark 5.1 From Remark 4.2, one obtains that $P_{S_{1}}^{3}$ and $P_{S_{2}}^{2}$ cannot be the regular equilibria at the same time. Therefore, for the positive equilibria $P_{S_{1}}^{3}$ and $P_{S_{2}}^{3}$, we have the following conclusions.

(i) If $I_{th}<I_{S_{2}}^{⁎}<I_{S_{1}}^{⁎}$, then $P_{S_{1}}^{3}$ is a virtual equilibrium, while $P_{S_{2}}^{2}$ is a regular equilibrium;
(ii) if $I_{S_{2}}^{⁎}<I_{th}<I_{S_{1}}^{⁎}$, then $P_{S_{1}}^{3}$ and $P_{S_{2}}^{2}$ are both virtual equilibria;
(iii) if $I_{S_{2}}^{⁎}<I_{S_{1}}^{⁎}<I_{th}$, then $P_{S_{1}}^{3}$ is a regular equilibrium, while $P_{S_{3}}^{2}$ is a virtual equilibrium.

**Figure 2:**
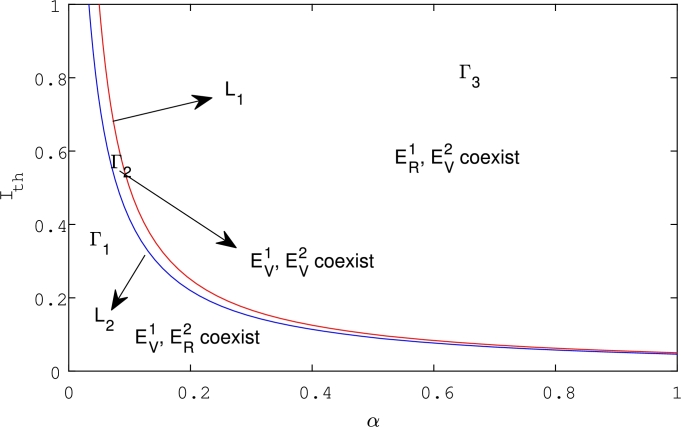
Bifurcation set for system (2) with respect to *α* and *I*_*th*_. The parameters are fixed as follows: *β* = 0.2, Λ = 0.8, *μ* = 0.05, and *q* = 0.2.

All the cases listed above can be viewed in Fig. 2, where we define two curves

$$L_{1}=\left\{(\alpha ,I_{th})|I_{S_{2}}^{⁎}=\frac{\mu }{\alpha }\right\},L_{2}=\left\{(a,I_{th})|I_{S_{2}}^{⁎}=\frac{\mu R_{S_{2}}^{⁎}}{\alpha R_{S_{2}}^{⁎}+q}\right\}.$$

Fig. 2 shows that with different the threshold value of $I_{th}$ and the parameter *α*, the equilibria $P_{S_{1}}^{3}$ and $P_{S_{2}}^{2}$ can switch between the regular and the virtual equilibrium. Furthermore, it shows that the rumor spreading equilibrium constantly decreases as the parameter *α* increases.

### 5.2 Existence of a sliding domain

It is well known that if two subsystems of the system (2) are oriented toward each other in $\Sigma_{s}$, a sliding domain may occur. Next, the sliding mode dynamics of the Filippov system (2) will be derived using the equivalent control method [27].

The following is a sufficient requirement for the existence of a sliding mode on a discontinuous surface

$$H(z)\frac{\partial H}{\partial z}\frac{dz}{dt}<0.$$

From system (1), we obtain

$$H(z)\frac{\partial H}{\partial z}\frac{dz}{dt}=(I-I_{th})\left(\begin{array}{ccc} 0\; & 1\; & 0 \end{array}\right)\left(\begin{array}{c} \Lambda -\beta SI-\mu S\;\\ \beta SI-\alpha IR-\mu I-\varepsilon qI\;\\ \alpha IR-\mu R+\varepsilon qI\; \end{array}\right)=(I-I_{th})(\beta SI-\alpha IR-\mu I-\varepsilon qI).$$

When ε=0, $I-I_{th}<0$, there must be βSI−αIR−μI>0 in order that $H(z)\frac{\partial H}{\partial t}<0$. As a result, we have βS−αR>μ. When ε=1, there must be βSI−αIR−μI−qI<0. So, βS−αR<μ+q. So, the sliding segment of Filippov system (2) can be written as

$$\Sigma_{S}=\left\{{\left(S,I,R\right)}^{T}\in R_{+}^{3}|I=I_{th},\mu \leq \beta S-\alpha R\leq \mu +q\right\}$$

### 5.3 Sliding mode dynamics

Using the Utkin equivalent control method [27], we can derive the dynamics of the Filippov system (2) on the boundary $\Sigma_{s}$.

More specifically, H(z)=0 and

$$\frac{\partial H}{\partial t}=\beta SI-\alpha IR-\mu I-\varepsilon qI=0\;\text{with}\;I=I_{th}.$$

Therefore, we obtain

$$\varepsilon =\frac{\beta S-\alpha R-\mu }{q}.$$

Putting *ε* into the second equation of system (1) gives us

(23) $$\left\{ \begin{array}{c} \frac{dS}{\mathrm{dt}}=\Lambda -\beta SI_{th}-\mu S,\;\\ \frac{dR}{\mathrm{dt}}=I_{th}\beta S-\mu R(t-\tau )-\mu I_{th}.\; \end{array}\right.$$

System (23), which has a unique pseudo-equilibrium $E_{P}(S_{p},I_{th},R_{p})$, can be used to figure out the dynamics of $\Sigma_{S}$, where

$$S_{p}=\frac{\Lambda }{\beta I_{th}+\mu },R_{p}=\frac{\Lambda \beta I_{th}-\mu I_{th}(\beta I_{th}+\mu )}{\mu (\beta I_{th}+\mu )}.$$

Theorem 5.1 *If the pseudo-equilibrium* $E_{P}$ *exists, then it is locally asymptotically stable.*

Proof Linearizing system (23) at the pseudo-equilibrium $E_{p}$, we obtain the characteristic equation as follows

$$(\lambda +\beta I_{th}+\mu )(\lambda +\mu e^{-\lambda \tau })=0.$$

Obviously, $\lambda =-\beta I_{th}-\mu <0$. Obviously, all roots of $\lambda +\mu e^{-\lambda \tau }=0$ have negative real parts. Therefore, the pseudo-equilibrium $E_{p}$ is locally asymptotically stable with all τ≥0. □

Remark 5.2 The existence of sliding mode is very important for discontinuous control. We can make the system converge to any point in sliding domain $\Sigma_{S}$ by selecting some value of $I_{th}$. From the realistic perspective, it implies that we can control the rumor spread in a small range by selecting the value of $I_{th}$.

## 6 Numerical simulations

In this part, we demonstrate our theoretical results using numerical simulations of a few situations.

### 6.1 The stability of $P_{S_{1}}^{1}$ and $P_{S_{1}}^{2}$

We choose the parameters as follows.

α=0.4,β=0.3,Λ=0.5,μ=0.5,q=0.6.

**Figure 3:**
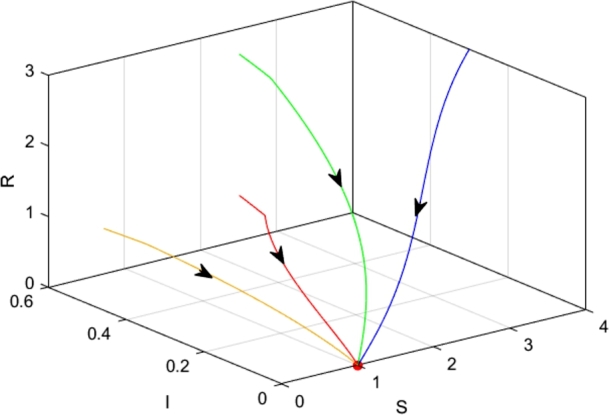
$P_{S_{1}}^{1}$ is locally asymptotically stable.

It is easily obtained that $\Lambda \beta <\mu^{2}$, according to Theorem 4.1, the equilibrium $P_{S_{1}}^{1}=(\frac{\Lambda }{\mu },0,0)$ is locally asymptotically stable (Fig. 3), meaning that rumor is wiped out in the social realm.

**Figure 4:**
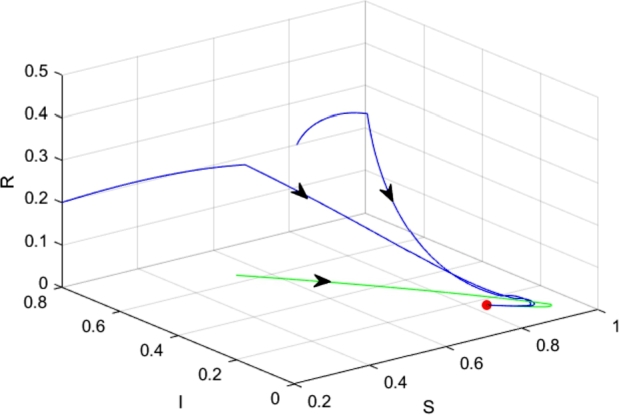
$P_{S_{1}}^{2}$ is locally asymptotically stable.

If we change *β* from 0.3 to 0.6, then $\mu^{2}<\Lambda \beta <\mu^{2}+\frac{\mu^{2}\beta }{\alpha }$, according to Theorem 4.1, the equilibrium $P_{S_{1}}^{1}=(\frac{A}{\mu },0,0)$ is unstable, and the equilibrium $P_{S_{1}}^{2}=(\frac{\mu }{\beta },\frac{\Lambda \beta -\mu^{2}}{\beta \mu },0)$ is locally asymptotically stable for any τ≥0 (Fig. 4). It suggests that rumors propagate steadily in the social domain.

From the above, we can obtain that *β* has a great impact on the dynamics of system (1), and it has a threshold value $\beta_{c}$. If $\beta <\beta_{c}$, then rumor cannot spread. While, if $\beta >\beta_{c}$, then rumors were spread. This indicates that when the link between rumor-susceptible and rumor-spreading persons grows, it becomes easier for rumors to spread. In this context, the spread of rumors poses a significant threat to social stability.

### 6.2 The existence of the sliding domain

We assume the parameter values as follows.

α=0.4,β=0.6,Λ=0.7,μ=0.1,q=0.6.

Therefore, we obtain that $P_{S_{1}}^{3}=(2.8000,0.2500,3.9500)$, and $P_{S_{2}}^{2}=(3.4306,0.1734,3.3960)$.

**Figure 5:**
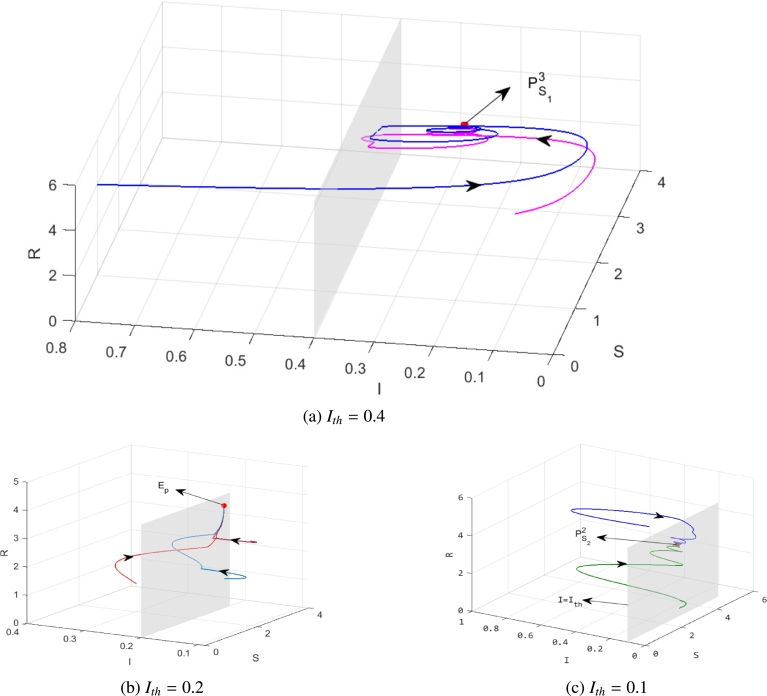
The dynamics of system (2) with different *I*_*th*_. (a) *I*_*th*_ = 0.4; (b) *I*_*th*_ = 0.2; (c) *I*_*th*_ = 0.1.

First, we let the parameter $I_{th}=0.4$, the equilibrium $P_{S_{1}}^{3}$ is locally asymptotically stable, and $P_{S_{2}}^{2}$ is a virtual equilibrium, see Fig. 5(a).

Then, we change the parameter $I_{th}$ to 0.2, which satisfies $0.1734<I_{th}<0.2500$. Therefore, the equilibria $P_{S_{1}}^{3}$ and $P_{S_{2}}^{2}$ are both virtual equilibria, and system (2) has a pseudo-equilibrium $E_{P}=(3.1818,0.2,3.9168)$, which is stable, see Fig. 5(b). In fact, for all $0.1734<I_{th}<0.2500$, the system has a pseudo-equilibrium $E_{p}=(S_{p},I_{p},R_{p})$, where

$$S_{p}=\frac{\Lambda }{\beta I_{th}+\mu },I_{p}=I_{th},R_{p}=\frac{\Lambda \beta I_{th}-\mu I_{th}(\beta I_{th}+\mu )}{\mu (\beta I_{th}+\mu )}.$$

From Theorem 5.1, we know that it is locally asymptotically stable if it exists.

Furthermore, we let $I_{th}=0.4$, the equilibrium $P_{S_{1}}^{3}$ is a virtual equilibrium, while $P_{S_{2}}^{2}$ is a regular equilibrium, which is stable, see Fig. 5(c).

From a realistic perspective, we choose some $I_{th}$ such that we can keep the spread of rumors in a small range through the discontinuous control.

### 6.3 The effect of delay *τ*

In the following, we discuss the effect of *τ*. We let the parameters be same as the above. By a direction calculation, we obtain that Eq. (7) has two positive roots: $\omega_{1}=0.5610$ and $\omega_{2}=0.3699$. Substituting the system parameters into (9) yields the critical values of time delays *τ* as the following:

$$\tau_{1}^{j}=1.3845,12.5853,23.7860,34.9868,46.1875,\cdots ,$$

and

$$\tau_{2}^{j}=5.9800,22.9664,39.9528,56.9391,73.9255,\cdots .$$

In addition, from (20), we obtain

dReλ(τ)dτ|τ=τ1(j),λ=iω1=147.1872>0,dReλ(τ)dτ|τ=τ2(j),λ=iω2=−14.3336<0.

**Figure 6:**
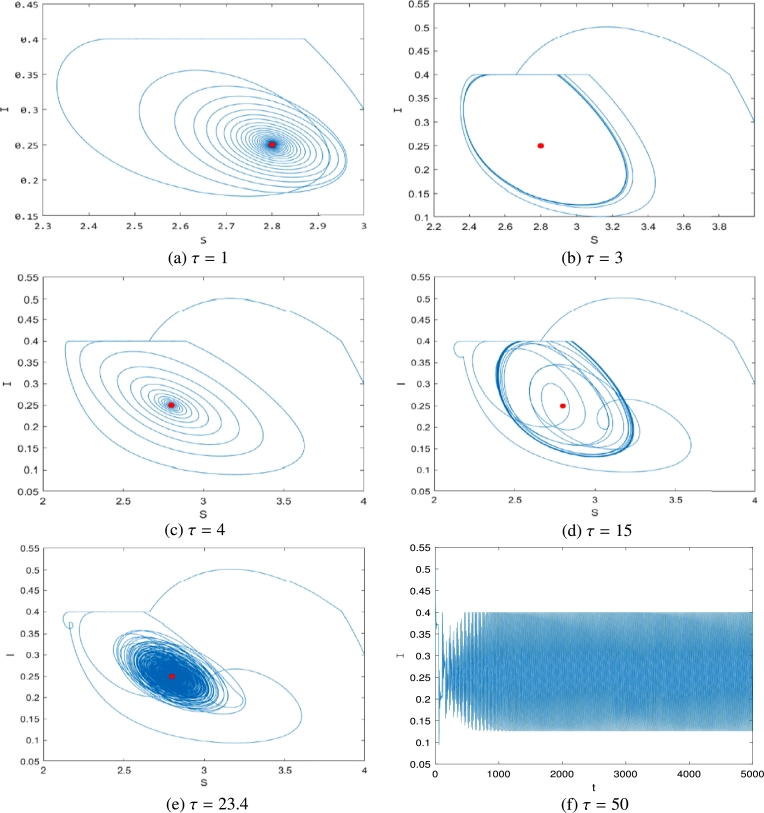
Time history with varying time delay *τ* with *I*_*th*_ = 0.4. (a) *τ* = 1; (b) *τ* = 3; (c) *τ* = 4; (d) *τ* = 15; (e) *τ* = 23.4; (f) *τ* = 50.

When $\tau =\tau_{1}^{j}$, a pair of eigenvalues crosses the imaginary axis from left to right. Fig. 6 shows the delay time histories from different locations with $I_{th}=0.4$, indicating that $P_{S_{1}}^{3}$ is a regular equilibrium. When $\tau \in [0,\tau_{1}^{1})⋃(\tau_{2}^{1},\tau_{1}^{2})⋃(\tau_{2}^{2},\tau_{1}^{3})$, the equilibrium of the system (2) is asymptotically stable, but it becomes unstable when $\tau \in (\tau_{1}^{1},\tau_{2}^{1})⋃(\tau_{1}^{2},\tau_{2}^{2})⋃(\tau_{1}^{3},+\infty )$. In other words, time delay causes the system (2) to display the phenomena of many switching events, where the state of the system changes from stable to unstable and back again. The system (2) is ultimately unstable at the equilibrium position $P_{S_{1}}^{3}$.

Fig. 6(a), (c), (e) show that the rumor-propagating individuals converge to a positive number, implying that rumors spread in the social realm in a stable state. Fig. 6(b), (d) show that the rumor-propagating individuals maintain a fluctuating state, which means that the rumor continues to break out in stages. Fig. 6(f) shows that the rumor in the system (1) will continue to erupt periodically.

**Figure 7:**
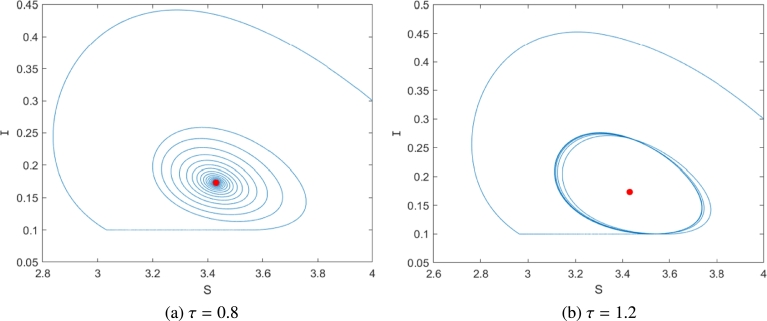
Time history with varying time delay *τ* with *I*_*th*_ = 0.4. (a) *τ* = 0.8; (b) *τ* = 1.2.

Similarly, for subsystem $S_{2}$, Eq. (18) has only a positive root ω=0.8167. So, according to (19), we have $\tau_{2}^{0}=1.0994$. Therefore, when τ=0.8∈[0,1.0994) the equilibrium $P_{S_{2}}^{2}$ is locally asymptotically stable (Fig. 7(a)), and when τ=1.2∈(1.0994,+∞), see Fig. 7(b), where $I_{th}=0.1$. That is, above the critical value τ=1.2, rumors will continue to erupt periodically.

**Figure 8:**
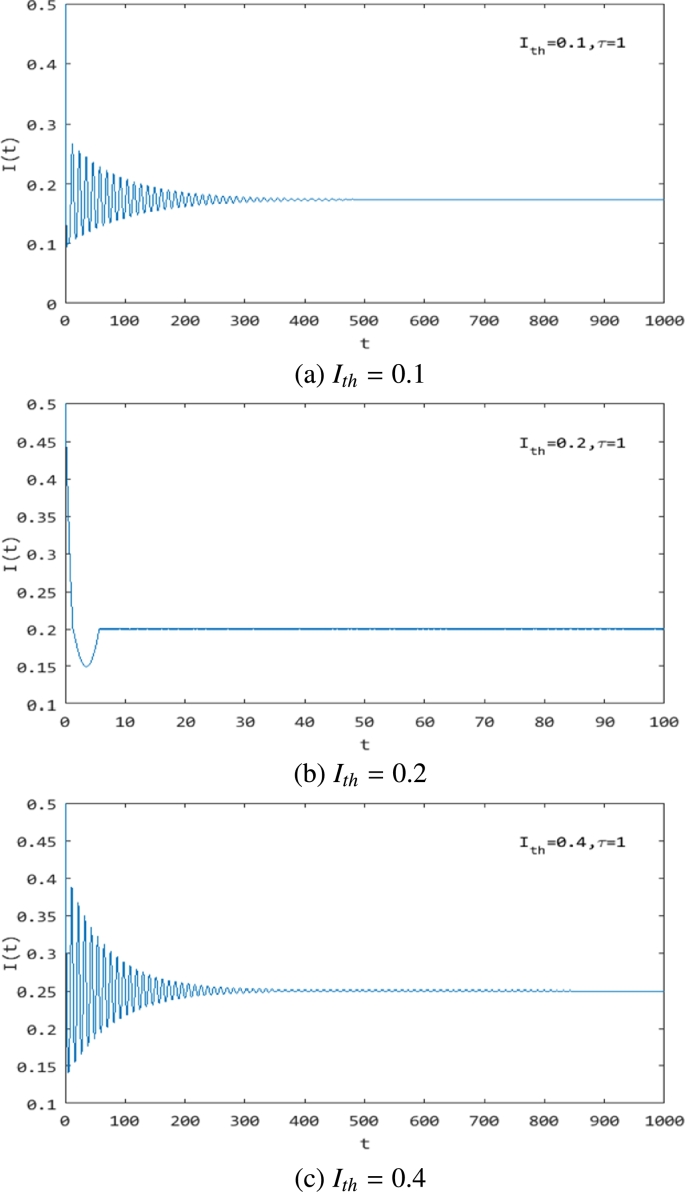
The change in the rumor-propagating individuals with different *I*_*th*_ and *τ* = 1. (a) *I*_*th*_ = 0.1; (b) *I*_*th*_ = 0.2; (c) *I*_*th*_ = 0.4.

**Figure 9:**
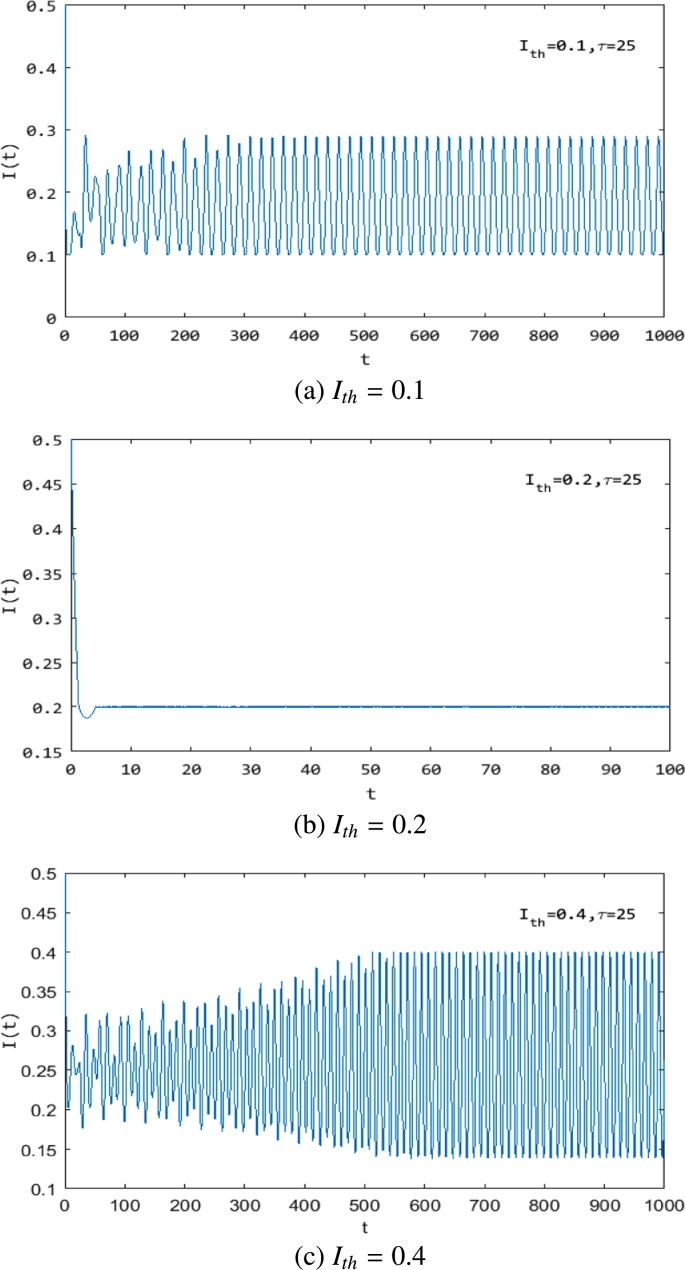
The change in the rumor-propagating individuals with different *I*_*th*_ and *τ* = 25. (a) *I*_*th*_ = 0.1; (b) *I*_*th*_ = 0.2; (c) *I*_*th*_ = 0.4.

**Figure 10:**
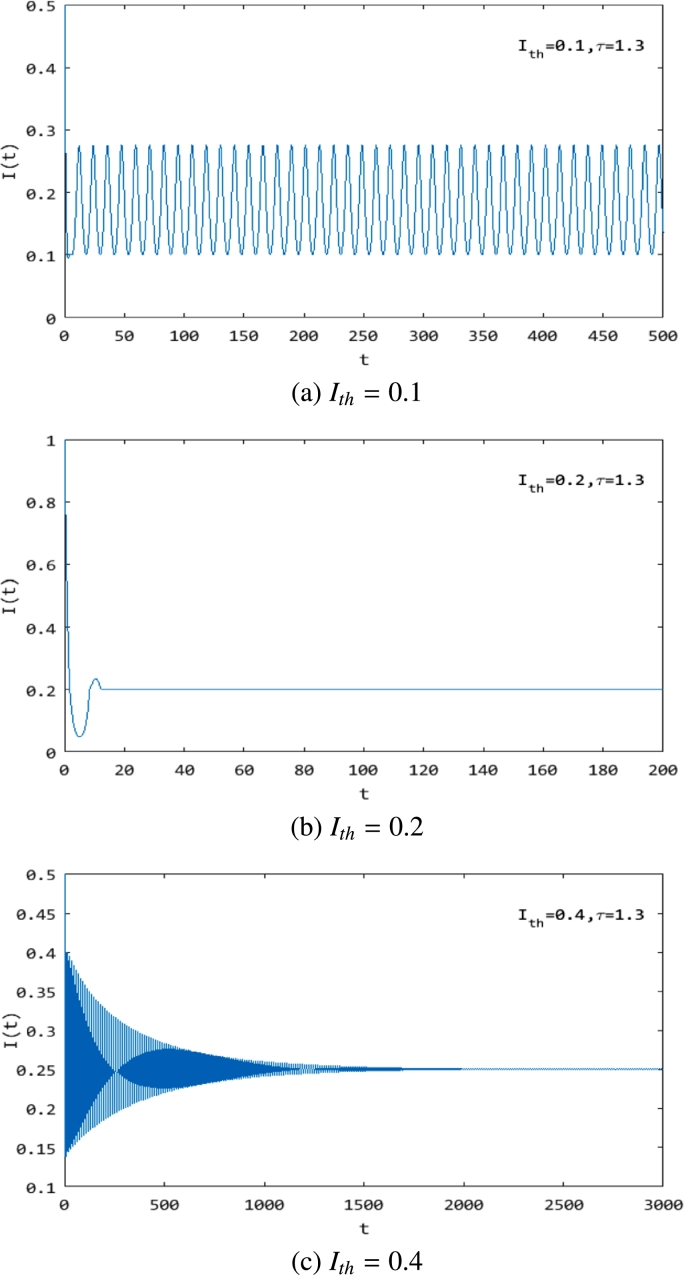
The change in the rumor-propagating individuals with different *I*_*th*_ and *τ* = 1.3. (a) *I*_*th*_ = 0.1; (b) *I*_*th*_ = 0.2; (c) *I*_*th*_ = 0.4.

From Fig. 6 and Fig. 7, it is easily obtained that we can control the rumor in a certain range by choosing the value of $I_{th}$. We show these in Fig. 8-Fig. 10, where $I_{th}=0.1$ (Fig. 8(a), Fig. 9(a), Fig. 10(a)), $I_{th}=0.2$ (Fig. 8(b), Fig. 9(b), Fig. 10(b)) and $I_{th}=0.4$ (Fig. 8(c), Fig. 9(c), Fig. 10(c)). They show that with the increase in $I_{th}$, the maximum value of the rumor-propagating individuals becomes larger. That is to say, we can control the rumor in a smaller range by choosing $I_{th}$. From Fig. 9 and Fig. 10, we also obtain that the threshold value $I_{th}$ can change the stability of system (1). In addition, Fig. 8(b), Fig. 9(b), and Fig. 10(b) show that the system converges to the equilibrium in finite time. Indeed, this is a unique property of a discontinuous system that a smooth ODE system can not have, and using this feature, we can quickly and effectively control the spread of rumors.

### 6.4 Some comparison results

To demonstrate the significance of the discontinuous control strategy in system (1), we compare it to Ref. [18], which has a similar system to system (1) but with a saturated control function $\frac{\beta_{1}I}{1+\alpha_{1}I}$.

**Figure 11:**
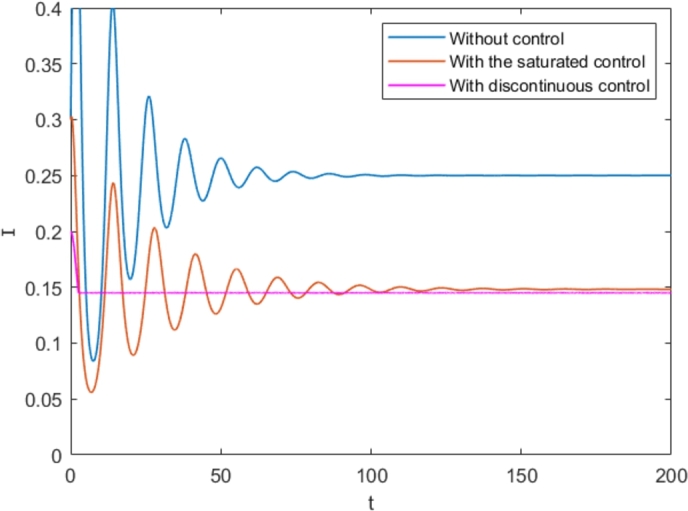
Dynamics of system (1) without any control strategies, with the saturated control and with the discontinuous control with *I*_*th*_ = 0.145 and *τ* = 0.

We choose the following parameters

$$\alpha =0.4,\beta =0.5,\Lambda =0.6,\mu =0.1,\alpha_{1}=0.4,\beta_{1}=0.7,\tau =0,$$

which are selected in Ref. [18]. Then, dynamics of system (1) without control strategies, with the saturated control and with the discontinuous control are shown in Fig. 11. From it, we obtain that by the effect of controller, the number of people spreading rumors has decreased, and the rumor spreaders are stable to a lower level under the discontinuous control. In addition, we also observed that when compared to saturation control, the system converges to equilibrium faster with discontinuous control. It demonstrates that by using a discontinuous controller, we can control the spread of rumors more quickly.

**Figure 12:**
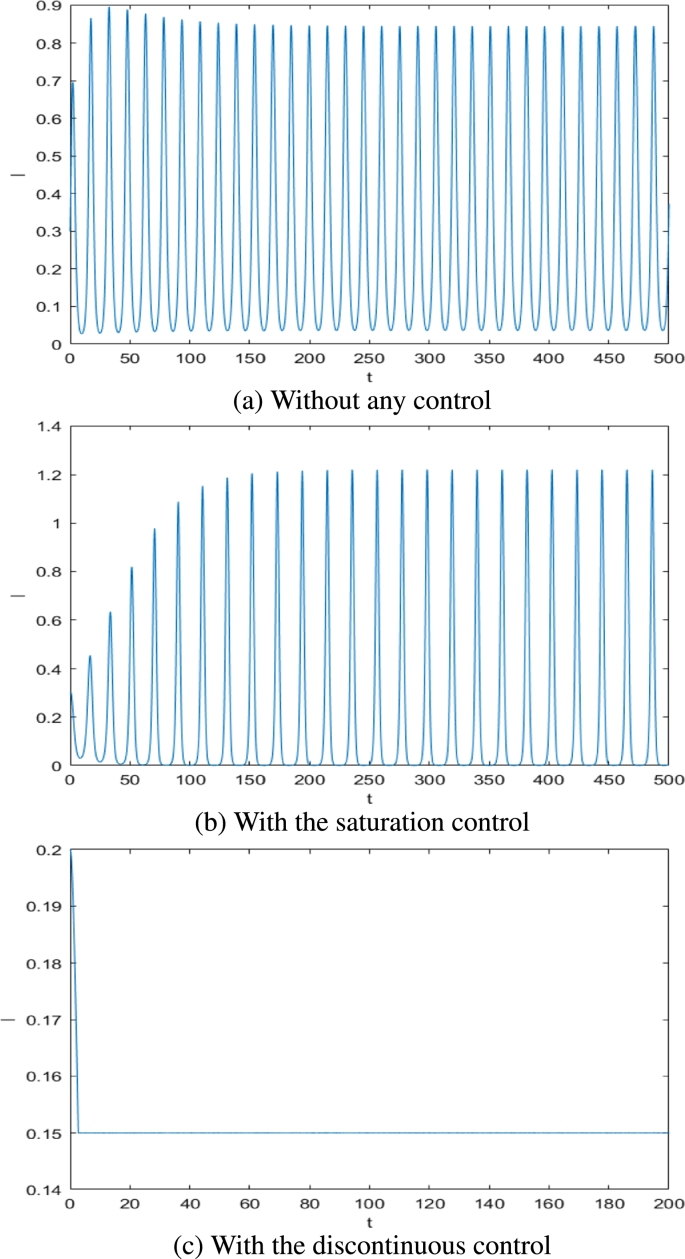
Dynamics of system (1) without control strategies, with the saturated control and with the discontinuous control when *τ* = 2, *I*_*th*_ = 0.15. (a) Without any control; (b) With the saturation control; (c) With the discontinuous control.

Now we let τ>0, from Ref. [18] we know that when *τ* above a threshold value system will be in a state of periodic oscillation, which makes it hard to stop the spread of the rumors. However, with a discontinuous control we can adjust the value of $I_{th}$ to control rumor quickly. Fig. 12 (a) shows that when τ=2, system is in a periodic oscillation. With the saturation control system is still in the periodic oscillation (see Fig. 12(b)), which means that saturation control fails to effectively control the spread of rumors. However, Fig. 12(c) shows that rumors were quickly brought under control with discontinuous control.

## 7 Conclusions

In this study, we examine a model for the spread of rumors that involves a threshold that can be set arbitrarily high or low. We first performed a dynamic analysis of the two parts of the system and derived the necessary conditions for the local stability of the equilibrium states. Hopf bifurcation at the stable equilibrium was investigated with *τ* as the bifurcation parameter. Theoretical results and simulations showed that *τ* is what causes the model to switch between stable and unstable states, and Hopf bifurcation happens when *τ* goes above a threshold.

We also studied the sliding domain and its dynamics, including the existence of the tangent equilibrium, boundary equilibrium, regular equilibrium, and the stability of the pseudo-equilibrium.

Indeed, the situation of people in the social world may become more complicated when rumors break out, and this can influence the rumor's spread. We plan to further explore and improve the model of rumor spread and to provide more practical control techniques in the future. In addition, we mentioned how the complex network theory became more frequently used in the research of rumor spread thanks to Watts's WS small world network model [28] and Barabasi's BA scale-free model. It, we'll think about how to stop rumors from spreading on social media and analyze the techniques that people use to do so.

## Declarations

### Author contribution statement

Chunru Li: Conceived and designed the experiments; Analyzed and interpreted the data; Wrote the paper. Zujun Ma: Conceived and designed the experiments; Contributed reagents, materials, analysis tools or data. Yuanyuan Wang: Performed the experiments; Analyzed and interpreted the data.

### Funding statement

Zujun Man was supported by 10.13039/501100001809National Natural Science Foundation of China [71672154].

### Data availability statement

Data will be made available on request.

### Declaration of interests statement

The authors declare no conflict of interest.

### Additional information

No additional information is available for this paper.
